# Characteristics of peripheral blood Gamma-glutamyl transferase in different liver diseases

**DOI:** 10.1097/MD.0000000000028443

**Published:** 2022-01-07

**Authors:** Mindan Xing, Min Gao, Jia Li, Ping Han, Ling Mei, Lili Zhao

**Affiliations:** a Nankai University School of Medicine, No. 94 Weijin Road, Tianjin, China; b Tianjin Second People's Hospital, Tianjin Institute of Hepatology, No. 7, Sudi South Road, Nankai District, Tianjin, China; c Graduate School, Tianjin Medical University, No. 22 Qixiangtai Road, Tianjin, China.

**Keywords:** alcoholic liver disease, drug-induced liver injury, Gamma-glutamyl transferase, non-alcoholic fatty liver disease, primary biliary cholangitis

## Abstract

Gamma-glutamyl transferase (GGT) is a marker of oxidative stress and cholestasis. Because of its low specificity, clinicians usually ignore its diagnostic value.

To compare and analyze the clinical features of GGT in primary biliary cholangitis (PBC), drug-induced liver injury (DILI), alcoholic liver disease (ALD), and non-alcoholic fatty liver disease (NAFLD) from the perspective of different causes instead of the severity of the disease.

We observed the distribution characteristics and the rate of abnormality of GGT in the above 4 diseases. The relationship between GGT and alanine aminotransferase (ALT), aspartate transaminase (AST), alkaline phosphatase (ALP), total serum bilirubin, triglyceride (TG), total cholesterol (TC), low-density lipoprotein cholesterol, high-density lipoprotein cholesterol was analyzed using *Spearman* correlation.

The highest level of GGT was up to 1000.00 to 2000.00 U/L in PBC and DILI, and the highest level of GGT was more than 2000.00 U/L in ALD, yet the difference was not statistically significant (*P* > .05). The highest level of GGT was only about 200.00 U/L in NAFLD and was the lowest in 4 liver diseases. Also, GGT was positively correlated with ALP, TC in PBC and DILI. Also, in ALD, GGT was positively correlated with ALT, AST, ALP, TG, and TC. In NAFLD, GGT was positively correlated with ALT, AST, and TG.

The abnormal GGT in PBC and cholestasis DILI was associated with cholestasis; in ALD, it was associated with oxidative stress and cholestasis, and in NAFLD, it was associated with oxidative stress. GGT levels had different characteristics in different liver diseases, which were closely related to the pathogenesis of liver diseases.

## 1 Introduction

Gamma-glutamyl transferase (GGT) is an enzyme that binds to the plasma membrane^[1,2]^ and is expressed in the kidney, liver, spleen, pancreas, small intestine, and so on. Its content is the highest in the kidney, followed by the liver.^[3,4]^ Renal disease rarely causes the increase of serum GGT; thus, such an increase is more common in liver disease.^[3,5]^ GGT in the liver is mainly located in the capillary side of the liver cells and the membrane of the bile duct epithelial cells. Hyper-synthesis in the liver, obstruction of bile excretion, and injury and hyperplasia of the bile duct epithelium can cause elevated serum GGT, which in turn can be used to help diagnose cholestatic liver disease.^[5]^

The main role of GGT is to metabolize L-glutathione (GSH) on the lateral side of the plasma membrane, which can convert amino acids into the precursor of GSH, and promote the binding of some endogenous substances such as leukotriene, prostaglandin, and exogenous substances to GSH, thus accelerating cell metabolism and excretion of exogenous substances.^[6]^ In addition, GGT participates in producing reactive oxygen species (ROS), causing DNA damage and regulating cell proliferation and apoptosis through pro-oxidation. As a result, serum GGT is a marker of oxidative stress, which is involved in redox regulation and inflammatory response, and is related to the development of vascular disease, metabolic syndrome, diabetes, cancer, and others.^[7]^

**Figure 1:**
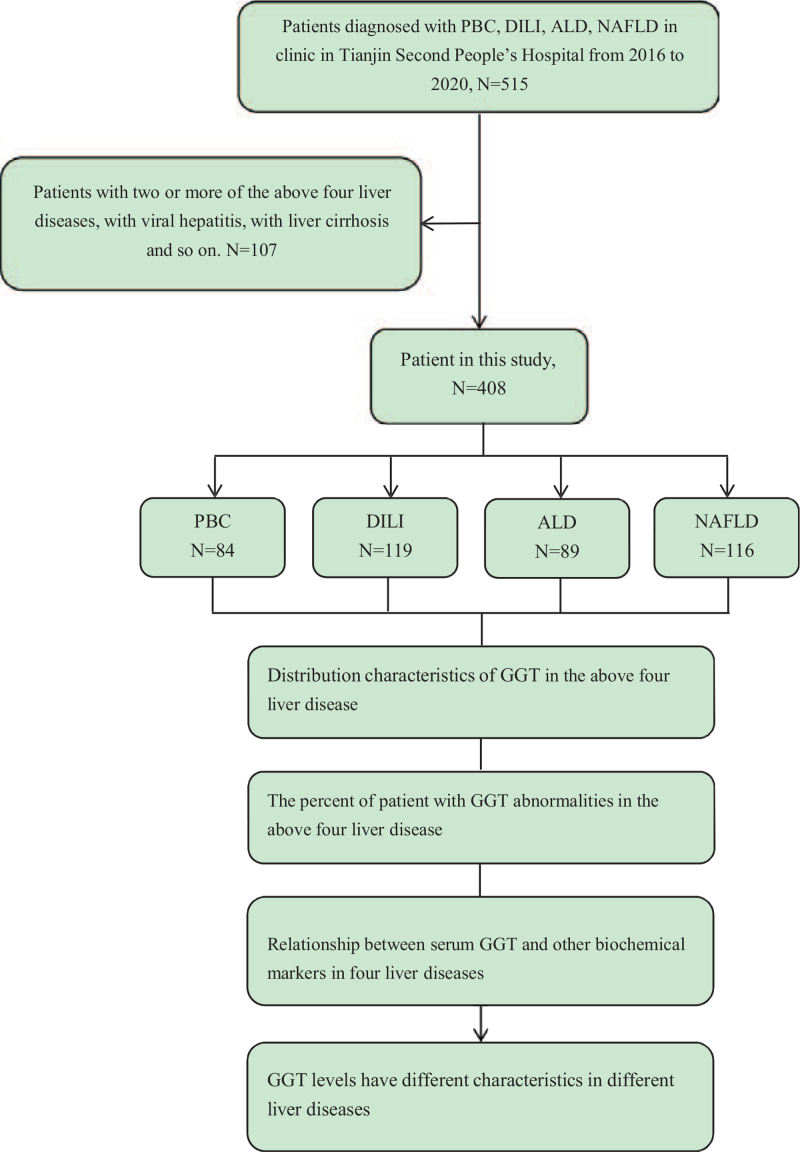
Flowchart for patient enrollment was shown. ALD = alcoholic liver disease, DILI = drug-induced liver injury, GGT = Gamma-glutamyl transferase, NAFLD = non-alcoholic fatty liver disease, PBC = primary biliary cholangitis.

While GGT can be used as a marker of bile duct injury and oxidative stress to help diagnose hepatobiliary diseases,^[7,8]^ clinicians usually ignore its significance due to the lack of specificity. In fact, elevated GGT is commonly found in many liver diseases, and understanding the characteristics of GGT in different liver diseases may be helpful to understand the pathogenesis of liver diseases further, as well as to select therapeutic targets. Primary biliary cholangitis (PBC), drug-induced liver injury (DILI), alcoholic liver disease (ALD), and non-alcoholic fatty liver disease (NAFLD) are the main causes of the abnormal GGT in clinic. In this study, the clinical characteristics of serum GGT in the above 4 liver diseases were observed and analyzed in order to provide clinical evidence for improving the diagnostic value of serum GGT and understanding the pathogenesis of liver diseases (the flowchart is shown in Fig. 1).

## 2 Methods

### 2.1 Case collection

A total of 515 patients who were diagnosed with PBC, DILI, ALD, NAFLD in Tianjin's Second People's Hospital between January 2016 and September 2020 were included. The diagnosis of PBC was made in accordance with the “Primary Biliary Cholangitis: 2018 Practice Guidance from the American Association for the Study of Liver Diseases”^[9]^ The Roussel Uclaf Causality Assessment Method (RUCAM) recommended by the International Council of Medical Organizations (CIOMS) was used for DILI diagnostic criteria.^[10–12]^ ALD diagnostic criteria met “Diagnosis and Treatment of Alcohol-Associated Liver Diseases: 2019 Practice Guidance From the American Association for the Study of Liver Diseases”,^[13]^ and the diagnosis of NAFLD was in accordance with the “The diagnosis and management of nonalcoholic fatty liver disease: Practice guidance from the American Association for the Study of Liver Diseases”.^[14]^ Exclusion criteria were the following: patients with 2 or more of the 4 liver diseases listed above; patients with cytomegalovirus, Epstein-Barr virus, Coxsackie virus, viral hepatitis, autoimmune hepatitis, hereditary liver diseases, and biliary obstruction; patients with various causes of liver cirrhosis, liver cancer, liver failure, and the end-stage liver disease; patients with coronary heart disease, hypertension, and other cardiovascular and cerebrovascular diseases and diabetes. Following the screening, 408 patients were included in the study. All included patients signed informed consent.

This study protocol followed the ethical guidelines of the 1975 Declaration of Helsinki as confirmed by approval granted by the Ethics Committee of Tianjin Second People's Hospital.

### 2.2 Biochemical parameters

Fasting blood samples (2 mL) were taken within 24 hours after hospitalization to measure alanine aminotransferase (ALT), aspartate transaminase (AST), alkaline phosphatase (ALP), GGT, total serum bilirubin (TBIL), triglyceride (TG), and total cholesterol (TC), low-density lipoprotein cholesterol, and high-density lipoprotein cholesterol using an automatic biochemistry analyzer (Hitachi-7180). The reagents were purchased from Wako Pure Chemical Industries, Ltd. and BioSino Biotechnology & Science Inc.

### 2.3 Statistical methods

SPSS 23.0 (New York, NY) of International Business Machines Corporation (IBM) was used to analyze the data. Measurement data with normal distribution were expressed as “*Mean* *±* *SD*”, and *Independent-Sample t test* and *One-way ANOVA* were used for data analysis. Post-hoc tests were performed using the *Tukey-Kramer* test. Measurement data that did not conform to normal distribution were expressed as “*Median* (*P*_*25*_*, P*_*75*_)”, and *Mann-Whitney U* test, and non-parametric *Kruskal-Wallis H* test was used to analyze the data. *Spearman* correlation was used for correlation analysis.

## 3 Results

### 3.1 General information

Among 408 participants, 204 patients (50%) were male and 204 patients (50%) were female, with an average age of (48.71 ± 14.53 years). Among the participants, 84 patients (20.59%) were with PBC, 119 patients (29.17%) were with DILI (29.17%), 89 patients (21.81%) were with ALD, and 116 patients (28.43%) were with NAFLD. Table 1 shows the general information of the studied subjects.

**Table 1:** The general data of the studied subjects.

	PBC (n = 84)	DILI (n = 119)	ALD (n = 89)	NAFLD (n = 116)	*P*
Sex (male, %)	6^abc^ (7.14)	40^ade^ (33.61)	88^bdf^ (98.88)	70^cef^ (60.34)	<.001
Age	58.10 ± 10.07^abc^	48.64 ± 14.01^a^	49.38 ± 10.76^bf^	41.47 ± 14.72^cf^	<.001
ALT (U/L)	35.00^a^ (19.00, 50.00)	460.00^ade^ (208.00, 916.00)	71.00^d^ (30.50, 156.00)	64.00^e^ (26.00, 111.00)	<.001
AST (U/L)	41.00^a^ (31.00, 69.50)	282.00^ade^ (111.00, 564.00)	50.00^d^ (25.50, 138.00)	35.00^e^ (23.25, 62.50)	<.001
GGT (U/L)	180.00^c^ (125.00, 427.50)	218.00^e^ (125.00, 332.00)	317.00^f^ (94.50, 866.00)	62.00^cef^ (36.25, 105.00)	<.001
ALP (U/L)	179.00^bc^ (105.00, 257.00)	137.00^e^ (91.00, 184.50)	93.00^bf^ (68.50, 132.00)	72.00^cef^ (55.00, 92.50)	<.001
TBIL (μmol/L)	14.20^a^ (10.15, 20.05)	41.00^ade^ (17.60, 128.70)	20.00^df^ (11.75, 39.75)	12.70^ef^ (9.80, 16.10)	<.001
TG (mmol/L)	1.25^c^ (0.89, 1.88)	1.44 (1.02, 2.24)	1.43 (0.97, 2.43)	1.69^c^ (1.26, 2.42)	.014
TC (mmol/L)	5.57^abc^ (4.63, 6.97)	4.19^ade^ (3.61, 4.95)	4.84^bd^ (3.79, 5.92)	5.11^ce^ (4.18, 5.79)	<.001
LDL-C (mmol/L)	2.76^a^ (2.10, 3.55)	2.16^ade^ (1.70, 2.58)	2.58^d^ (1.95, 3.35)	2.97^e^ (2.20, 3.68)	<.001
HDL-C (mmol/L)	1.44^abc^ (1.02, 1.85)	1.22^a^ (0.80, 1.34)	1.01^b^ (0.78, 1.57)	1.13^c^ (1.06, 1.47)	<.001

### 3.2 The distribution characteristics and the rate abnormality of GGT in 4 liver diseases

**Figure 2:**
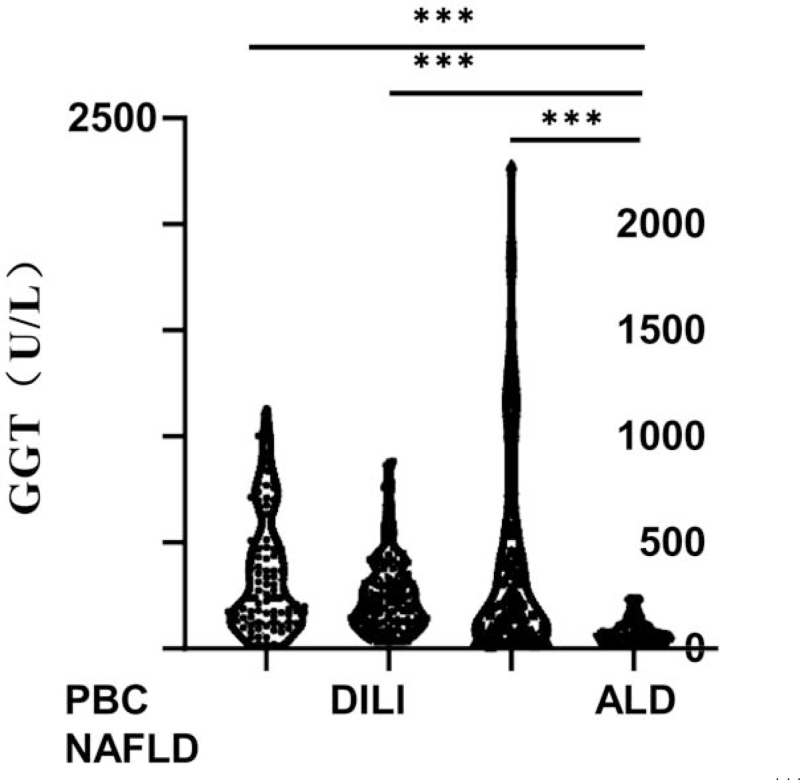
Distribution characteristics of GGT in different liver diseases. “^∗∗∗^” means *P* < .001. ALD = alcoholic liver disease, DILI = drug-induced liver injury, GGT = Gamma-glutamyl transferase, NAFLD = non-alcoholic fatty liver disease, PBC = primary biliary cholangitis.

In PBC patients, the median level of GGT was 180.00 U/L, and the highest level reached up to 1131.00 U/L; in DILI, the median level of GGT was 218.00 U/L, and the highest level reached up to 882.00 U/L; in ALD, the median level of GGT was 317.00 U/L, and the highest level reached up to 2280.00 U/L; in NAFLD, the median level of GGT was 62.00 U/L, while the highest level reached only to 239.00 U/L. Therefore, serum GGT level 62.00 (36.25, 105.00) U/L in NAFLD was the lowest among the 4 liver diseases, and the difference in the GGT level among the other 3 liver diseases was not statistically significant (Fig. 2).

There were 78 cases (78/84, 92.86%) with GGT abnormalities in the PBC patients, 114 cases (114/119, 95.80%) with GGT abnormalities in DILI, 69 cases (69/89, 77.53%) with GGT abnormalities in ALD, and 63 cases (63/116, 54.31%) with GGT abnormalities in NAFLD. The difference in abnormal rates of GGT was statistically significant among the 4 liver diseases (*P* < .001), and the highest rates of GGT anomalies were found in DILI and PBC (95.80% and 92.86%, respectively), and the lowest in NAFLD (54.31%) (Table 2).

**Table 2:** The percent of patient with GGT abnormalities in the above 4 liver disease.

	PBC (n = 84)	DILI (n = 119)	ALD (n = 89)	NAFLD (n = 116)	*P*
The percent of patient with GGT abnormalities					
Normal (N, %)	6 (7.14)^bc^	5 (4.20)^de^	20 (22.47)^bdf^	53 (45.69)^cef^	<.001
Abnormality (N, %)	78^bc^ (92.86)	114^de^ (95.80)	69^bdf^ (77.53)	63^cef^ (54.31)	

### 3.3 Biochemical markers associated with elevated GGT levels

As shown in Table 3, the GGT in PBC patients was positively correlated with ALT, AST, ALP, TBIL, and TC. The GGT in DILI patients was positively correlated with ALP and TC. The GGT in ALD patients was positively correlated with ALT, AST, ALP, TG, and TC. The GGT in NAFLD patients was positively correlated with ALT, AST, and TG.

**Table 3:** Correlation analysis of serum GGT and other biochemical indicators in 4 liver diseases.

	PBC		DILI		ALD		NAFLD	
	GGT(*r*)	*P*	GGT(*r*)	*P*	GGT(*r*)	*P*	GGT(*r*)	*P*
ALT	0.525	<.001	−0.067	.473	0.557	<.001	0.445	<.001
AST	0.461	.003	−0.080	.390	0.679	<.001	0.488	<.001
ALP	0.662	<.001	0.511	<.001	0.281	.008	0.151	.111
TBIL	0.350	.001	0.112	.228	0.194	.070	0.052	.583
TG	0.209	.064	−0.002	.986	0.407	.001	0.300	.001
TC	0.516	<.001	0.311	.001	0.431	<.001	0.008	.936
LDL-C	0.008	.951	0.133	.278	0.168	.169	−0.049	.644
HDL-C	0.105	.376	0.128	.220	−0.116	.341	0.011	.916

## 4 Discussion

PBC is an autoimmune disease characterized by non-suppurative inflammation of the bile duct, whose main clinical feature is intrahepatic cholestasis.^[9]^ In this study, most of the patients showed obvious ALP and GGT abnormalities, as well as ALT and AST mild abnormalities, which were consistent with the characteristics of PBC patients. The GGT in most PBC patients in this study was abnormal (92.86%) and positively correlated with ALP, which indicated that the main cause of GGT abnormalities in these patients was cholestasis. Besides, as cholesterol is the synthetic raw material of bile, which can be discharged into the intestine through the bile duct, PBC with cholestasis is often accompanied by cholesterol metabolism disorder.^[15]^ As a result, GGT in PBC patients was positively correlated with TC in this study, which is consistent with cholestasis.

Like PBC, the GGT level in 95.80% of DILI patients was abnormal and positively correlated with ALP and TC. As the main injury patterns of DILI include hepatocyte injury, cholestasis, and mixed type,^[10]^ the abnormal GGT in simple hepatocyte injury DILI patients is usually without cholestasis. Therefore, the correlation between GGT and cholestasis in DILI patients was weaker than in PBC patients. Still, in our previous study,^[16]^ we found that about 60% of DILI patients had cholestasis, so even if the simple hepatocyte injury affected the relationship between the GGT and cholestasis, there was also a high correlation between GGT level and cholestasis in DILI. The positive correlation between GGT and TC was also related to the presence of cholestasis in some patients with DILI.

The abnormal rate of GGT in ALD (77.53%) was lower than that in PBC and DILI, which may be caused by the genetic polymorphisms of enzymes associated with ethanol metabolism. With the different activity of metabolic enzymes, ethanol metabolism produces different ROS and oxidative stress levels, causing the different consumption of GSH. Therefore, the GGT in some active drinkers patients did not significantly increase even if they drank heavily in the clinic.^[17]^ Besides, GGT in ALD was positively correlated with ALT, AST, and ALP. The GGT in ALD patients may behave in 2 following ways: patient's GGT is raised without the elevated ALP. In this situation, the abnormal GGT is mainly related to oxidative stress caused by ethanol and its metabolites.^[18,19]^ In the second situation, GGT and ALP are elevated at the same time, and cholestasis occurs. When this happens, the abnormal GGT ensues due to oxidative stress caused by ethanol and cholestasis. The reported incidence of cholestatic ALD differs according to different inclusion criteria. The highest reported incidence was only 30%,^[20]^ which was significantly lower than that in PBC and DILI. Consequently, the correlation between GGT and ALP in ALD was weaker than that of PBC and DILI. In this study, ALD patients with cholestasis had more serious conditions, for example, ALT, AST, TBIL was higher than that in ALD patients without cholestasis (Table S1, Supplemental Digital Content), thus suggesting that such patients should be given early intervention and active treatment. Moreover, the metabolism of ethanol can also enhance fat mobilization and lipid metabolism disorder,^[19]^ so the GGT in ALD patients was also positively correlated with the TG, TC.

ALT and AST were normal or mildly abnormal in NAFLD patients, ALP and TBIL were completely normal, indicating that NAFLD patients usually have no cholestasis, and their abnormal GGT may not be associated with cholestasis. In addition, GGT was positively correlated with ALT, AST, TG in NAFLD. NAFLD patients have insulin resistance,^[21]^ which increases fat mobilization in adipose tissue and the uptake and synthesis of TG in the hepatocyte, thus causing the disorder of lipid metabolism and lipid peroxidation. GSH is the main thiol-antioxidant in the cell, and GGT is involved in GSH metabolism, so the level of GGT may increase with GSH consumption. Besides, GGT elevation induces GSH hydrolysis to cysteine glycine, where the latter can be oxidized to produce ROS, further causing liver inflammation.^[22]^ Therefore, the abnormal GGT in NAFLD patients is often accompanied by hepatocyte injury and lipid metabolism disorder.

We analyzed the characteristics of GGT in different liver diseases and provided evidence that further our understanding of the relationship between GGT abnormalities and pathogenesis in clinic. However, due to the limited number of cases, there was no stratified analysis of the patients included in this study, which resulted in the insufficient depth of understanding of the characteristics of GGT in each liver disease. Also, most patients did not undergo a liver pathology examination, so the relationship between GGT and liver pathology was not analyzed. In our next study, we plan to collect the information of the patient with liver pathology, analyze the relationship between GGT and liver pathology, and explore the mechanism of GGT elevation in different liver diseases from the basic mechanisms, as well as provide a scientific basis to further improve the clinical application value of GGT.

## 5 Conclusion

The abnormal GGT in PBC and cholestasis DILI was associated with cholestasis; in ALD, it was associated with both oxidative stress and cholestasis, and in NAFLD, it was associated with oxidative stress. GGT levels have different characteristics in different liver diseases, which were closely related to the pathogenesis of liver diseases.

## Acknowledgments

This work was supported by Tianjin Second People's Hospital in China. We thank Department of Pathology in Tianjin Second People's Hospital in China for help and Doctor Li Zhou, Jing Wang, and Chunyan Wang for help in collecting data.

## Author contributions

Mindan Xing and Min Gao substantially contributed to conception and design, acquisition of data, or analysis and interpretation of data; performing statistical analyses; drafting the article or revising it critically for important intellectual content; final approval of the version to be published. They participated to an equal extent in planning, analyzing, evaluating, and writing the paper.

Jia Li contributed to conception and design; drafting the article or revising it critically for important intellectual content; final approval of the version to be published; agreement to be accountable for all aspects of the work in ensuring that questions related to the accuracy or integrity of any part of the work are appropriately investigated and resolved.

Ping Han and Ling Mei contributions to acquisition of data.

Lili Zhao had analysis and interpretation of data; final approval of the version to be published.

**Conceptualization:** Jia Li.

**Data curation:** Mindan Xing, Min Gao, Jia Li, Ping Han, Ling Mei, Lili Zhao.

**Formal analysis:** Mindan Xing, Min Gao, Jia Li, Ping Han.

**Investigation:** Ling Mei.

**Methodology:** Mindan Xing, Jia Li, Ling Mei.

**Resources:** Jia Li, Ping Han, Lili Zhao.

**Software:** Mindan Xing.

**Supervision:** Min Gao, Jia Li.

**Writing – original draft:** Mindan Xing, Min Gao.

**Writing – review & editing:** Min Gao, Jia Li, Lili Zhao.

## Supplementary Material

Supplemental Digital Content
