# Supplementary material for: Characteristics of peripheral blood Gamma-glutamyl transferase in different liver diseases
Source: Medicine (Baltimore). 2022 Jan 7;101(1):e28443. doi: 10.1097/MD.0000000000028443 (PMC8735790; doi:10.1097/MD.0000000000028443)
Supplement: Supplemental Digital Content [file medi-101-e28443-s001.docx]

**Clinical characteristics of Gamma-glutamyl transferase in different liver diseases**

**Results**

Table1 Comparison of biochemical indices between ALD patients with GGT&ALP abnormalities at the same time and the other ALD patients

|  | GGT&ALP abnormalities  (n=24) | Others  (n=65) | *P* |
| --- | --- | --- | --- |
| ALT  (U/L) | 79.00  (37.00, 344.50) | 35.00  (21.00, 86.50) | 0.021 |
| AST  (U/L) | 83.00  (43.00, 189.50) | 38.00  (25.00, 77.50) | 0.003 |
| GGT  (U/L) | 540.00  (228.50, 1206.50) | 167.00  (57.50, 416.50) | 0.003 |
| ALP  (U/L) | 186.00  (143.00, 237.00) | 78.50  (65.00, 96.00) | <0.001 |
| TBIL  (umol/L) | 45.00  (24.00, 57.60) | 20.10  (13.65, 34.50) | 0.013 |
| TG (mmol/L) | 1.86  (1.29, 4.28) | 1.24  (0.92, 1.95) | 0.004 |
| TC (mmol/L) | 5.48  (3.99, 6.83) | 4.65  (3.74, 5.70) | 0.148 |
| LDL-C  (mmol/L) | 2.80  (1.76, 3.76) | 2.54  (1.99, 3.30) | 0.677 |
| HDL-C  (mmol/L) | 0.89  (0.50, 0.99) | 1.15  (0.90, 1.66) | 0.002 |

Note: GGT, Gamma-glutamyl transferase. PBC, primary biliary cholangitis. DILI, drug-induced liver injury. ALD, alcoholic liver disease. NAFLD, nonalcoholic fatty liver disease. ALT, alanine aminotransferase. AST, aspartate transaminase. ALP, alkaline phosphatase. TBIL, total serum bilirubin. TG, triglyceride. TC, total cholesterol. LDL-C, low-density lipoprotein cholesterol. HDL-C, high-density lipoprotein cholesterol.
